# Case Report: Low Rectal Cancer With Incidental Pelvic Solitary Kidney

**DOI:** 10.3389/fsurg.2021.819431

**Published:** 2022-02-03

**Authors:** Xiang Zhang, Chang Chen, Kexin Wang, Yong Dai, Yanlei Wang

**Affiliations:** Department of General Surgery, Qilu Hospital of Shandong University, Shandong, China

**Keywords:** rectal cancer, pelvic kidney, laparoscopy, case report, 3D reconstitution

## Abstract

**Purpose:**

Concurrence of pelvic solitary kidney and rectal cancer is a rare phenomenon. The presence of the kidney in a narrow pelvic cavity represents a great challenge for total mesenteric excision (TME) under laparoscopy.

**Methods:**

We reported a male patient with low rectal cancer and incidental pelvic solitary kidney and reviewed relevant literature.

**Results:**

The patient was successfully treated with laparoscopic surgery and was discharged on day 6 postoperatively without severe complications.

**Conclusion:**

This case suggests the feasibility of laparoscopic TME with pelvic solitary kidney in a certain male patient with rectal cancer and emphasizes the importance of comprehensive preoperative radiological evaluation, a multidiscipline team, and careful intraoperative dissection.

## Introduction

Pelvic kidney results from failure of the kidneys ascending to their usual position during the embryonic period. Most patients are asymptomatic and diagnosed accidentally. Pelvic solitary kidney occurs in 1:2,100–1:3,000 autopsies (1). The concurrence of pelvic kidney and rectal cancer is a rare phenomenon. In male patients, the presence of pelvic kidney makes the inherently narrow space of the pelvic cavity even more limited, posing a great challenge for total mesenteric excision (TME) under laparoscopy. Herein, we reported a male patient with simultaneous low rectal cancer and pelvic solitary kidney who was successfully treated with laparoscopic surgery.

## Case Presentation

**Figure 1:**
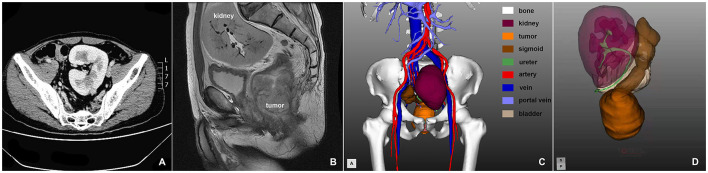
**(A)** Axial CT scan of the pelvic solitary kidney; **(B)** sagittal MRI scan of the pelvic solitary kidney and the rectum; **(C,D)** three-dimensional reconstruction of the pelvic organs.

A 49-year old male patient visited our hospital with 1 month of irregular defecation and hematochezia. He was then diagnosed with low rectal adenocarcinoma based on colonoscopy and pathological biopsy. A mass could be touched on the abdomen wall above the pubic symphysis by physical examination. Digital rectal examination showed a hard and fixed mass 1 cm above the anal verge; the upper verge of the mass could not be palpated. Contrast CT revealed no distal metastasis, and a previously unknown pelvic solitary kidney was identified. No seminal vesicle was observed. Three-dimensional CT reconstruction and pelvic MRI demonstrated that the pelvic kidney was 7.7 × 8.4 × 10 cm in size, and that 2/3 of the kidney was located in the pelvic cavity with the hilum facing toward the right common iliac artery (Figures 1A,B). Three separate renal arteries originated from the right common iliac artery and two separate renal veins drained into the left common iliac vein (Figure 1C). The ureter was short and tortuous (Figure 1D). The rectum and sigmoid colon were pushed to the right pelvic cavity. The patient had no awareness of the pelvic solitary kidney before, and the kidney function was normal after admission. Contrast pelvic MRI showed that the tumor had invaded the muscular layer; hence, preoperative tumor staging was cT2N0M0. The patient used to be healthy with no past medical history. After discussion with the multidisciplinary team (including a radiologist, oncologist, urologist, and pathologist), laparoscopic extralevator abdominoperineal excision (LELAPE) without neoadjuvant therapy was proposed. The patient agreed to our proposal and signed a consent form.

In the operating room, two senior surgical urologists were standing by during the whole surgery in case of additional injury of the urinary system. Under general anesthesia, the patient was first placed in the Trendelenburg position (30°) with a right lateral tilt (15–20°). The operator stood on the right side of the patient, the first assistant on the left, and the camera holder on the cranial side. The pneumoperitoneal pressure was set at 12 mmHg. A 10-mm trocar was inserted above the umbilicus as the observation site, a 12-mm or 10-mm main operating port was made about 5 cm below the umbilical level on the right midclavicular line, and a 5-mm assistant trocar was made at the umbilical level on the same line. At the planned site for sigmoid colon stoma, a 5-mm trocar was placed for the assistant which was later lengthened for colostomy. Another 5-mm trocar was made 2 cm above the pubic symphysis for assistance.

**Figure 2:**
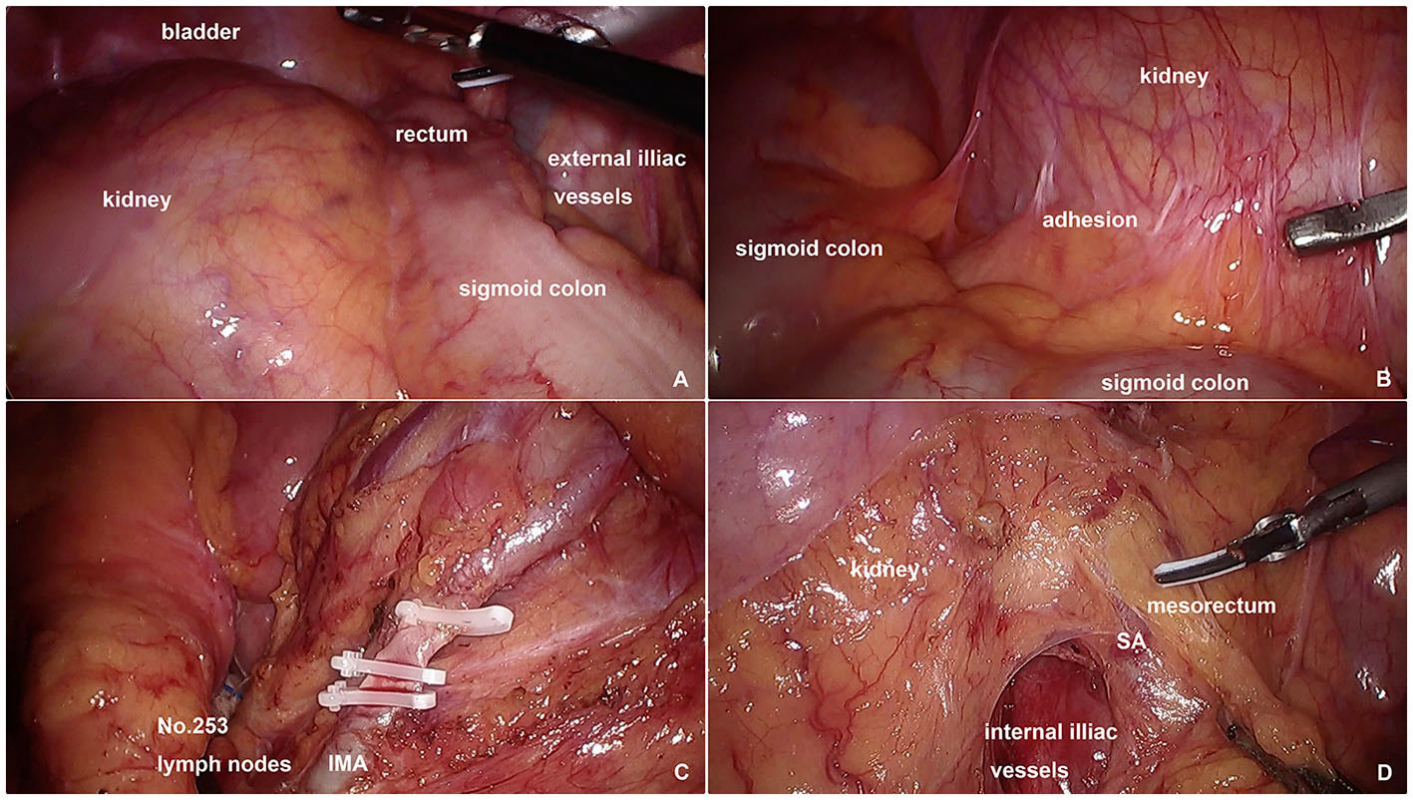
**(A–D)** Intraoperative images of abdominal dissection under laparoscopy.

**Figure 3:**
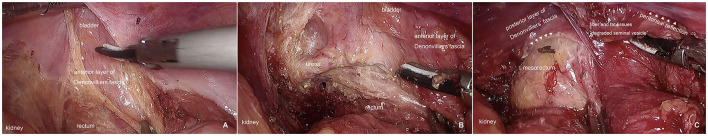
**(A–C)** Intraoperative images of pelvic dissection under laparoscopy.

At first, complete laparoscopic exploration was performed: the kidney was located in the pelvic cavity with the rectum and rectosigmoid pushed to the right (Figure 2A); adhesions were observed between the mesosigmoid and the peritoneum above the kidney as well as the mesoileum (Figure 2B); no tumor implants or occult liver lesions were identified. After adhesion lysis, a clear avascular plane between the mesosigmoid and the prehypogastric nerve fascia was identified and dissection proceeded medially along this plane to the left lateral peritoneal gutter. The inferior mesenteric artery (IMA) was ligated at about 1 cm from its origin after clearance of No.253 lymph nodes (Figure 2C). Pelvic dissection with TME began from the posterior and right sides and proceeded caudally. Despite the narrow pelvic space, the anatomy in these two sides was normal, and no difficulties in dissection were encountered. For the left side, the kidney and mesorectum were dissected under counter-traction using the superior rectal artery (SRA) as a landmark (Figure 2D). We were reminded of an increase in blood pressure by the anesthetist, so kidney retraction force was reduced to maintain the stability of blood pressure. The anterior dissection is the most challenging part. Usually, we cut the pelvic peritoneum approximately 1 cm above the peritoneal reflection. However, in this patient, without seminal vesicle being used as a landmark, we chose the peritoneal reflection as the cutting point, although we were still not able to recognize the Denonvilliers'fascia and accidentally entered an incorrect plane between the posterior wall of the bladder and the anterior layer of the Denonvilliers'fascia (Figure 3A). We had not realized this mistake until the ureter and left trigone of the bladder were exposed and identified (Figure 3B). Then, we transected the Denonvilliers'fascia and entered the correct surgical plane between the posterior layer of the Denonvilliers' fascia and the mesorectum (Figure 3C). The anterior dissection ceased after proceeding caudally for another 2 cm. The sigmoid mesocolon was trimmed to the edge of the sigmoid colon and then divided with a laparoscopic linear stapler intracorporeally. The end of the proximal colon was pulled out to create a colostomy.

**Figure 4:**
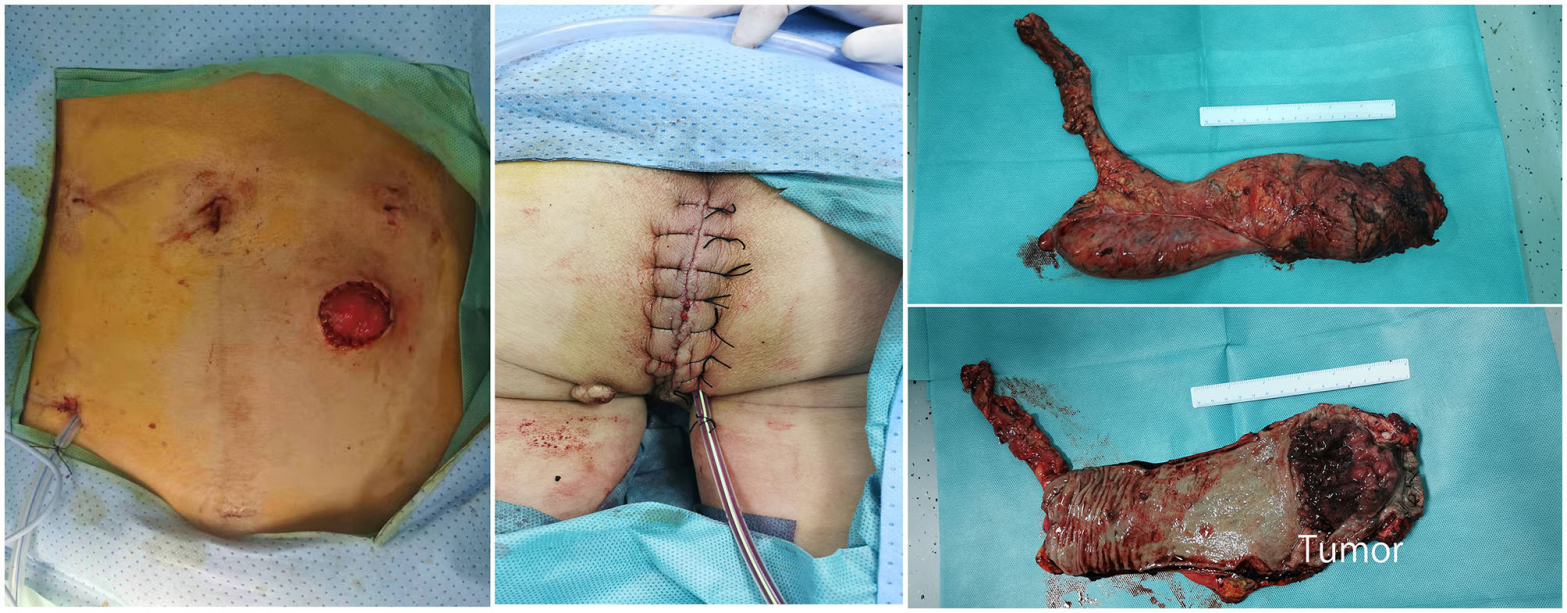
Postoperative images of abdominal and perineal incision and specimen.

The patient was then turned around into the prone jackknife position, and a purse-string suture was performed to close the anus. The perineal skin was incised from the coccyx to the perineum. Dissection followed the outer surface of the external sphincter muscle and the levator ani muscle, and the coccyx was removed. Posteriorly, the sacrococcygeal junction was divided to obtain access to the inner dissection plane. Afterward, the levator ani was divided laterally close to its origin. Then, the distal end of the sigmoid colon was pulled out of the pelvic cavity, and the anterior connection of the rectum and posterior wall of the prostate was exposed. Finally, the dissection was completed anteriorly with meticulous preservation of neurovascular bundles and the prostate. After the cylindrical specimen was removed, the ischiorectal fat and skin were closed. A presacral drainage tube was placed through the abdominal port incision, and a subcutaneous tube was placed in the perineal incision. The total operation time was 5 h and 10 min. Only one colostomy and several minor trocar incisions were presented in the abdominal wall without extra incisions (Figure 4). Postoperative pathology showed moderately differentiated adenocarcinoma with muscular layer invasion, no lymph node metastasis (pT2N0M0), and negative circumferential and longitudinal margins (R0). The patient was discharged on day 6 without any postoperative complications. No sign of recurrence was indicated by CT scan or blood test 6 months after the surgery.

## Discussion

Concurrence of pelvic kidney and colorectal cancer is a rare phenomenon. Nine case reports were found in the literature search (2–10), and only six full texts were available (2–7) (Table 1). Of the six reports, five patients were male and one patient was female. Pelvic solitary kidney was confirmed in one patient (5). Three patients were described with one kidney located in the pelvic cavity and and the other kidney located *in situ* (3, 4, 7). Two of the reports did not mention the situation of the other kidney (2, 6). Only one male patient with rectosigmoid colon cancer was successfully treated with laparoscopic surgery (4). For the rest, open surgery was initially chosen or intraoperative open surgery conversion was performed because of unexpected pelvic kidney or severe intraoperative complications. To our knowledge, the present case is the first report of a patient with simultaneous low rectal cancer and pelvic solitary kidney who was successfully treated with laparoscopic surgery.

**Table 1:** Case report of colorectal cancer with pelvic kidney.

**Year**	**Author**	**Journal**	**Nation**	**Gender**	**Age**	**Diagnosis**	**Left kidney**	**Right kidney**	**Operation**	**Complication**
1996	Malak B. Bokhari et al. (2).	J Surg Oncol	United States	Male	63	Rectal cancer	Not reported	In pelvis	Open surgery	Null
2005	Sakamoto K. et al. (5).	J Minim Access Surg	Japan	Male	55	Sigmoid colon cancer	In pelvis	Absent	Laparoscopic surgery and open surgery	Null
2018	Koki Takeda et al. (6).	Asian J Endosc Surg	Japan	Female	54	Rectal cancer	Not reported	In pelvis	Laparoscopic surgery with open surgery conversion	Intraoperative right renal artery ligation
2019	Hassan Moaiery et al. (3).	J Med Case Rep	Iran	Male	40	Rectal cancer	In situ	In pelvis	Open surgery	Postoperative anastomotic leak
2020	Katherine J. Zhu et al. (7).	ANZ J Surg	Australia	Male	65	Rectosigmoid colon cancer	In pelvis	In situ	Laparoscopic surgery and open surgery	Null
2021	Byung Kwan Park et al. (4).	Ann Coloproctol	Korea	Male	76	Rectosigmoid colon cancer	In pelvis	In situ	Laparoscopic surgery	Null

The kidneys are retroperitoneal organs. Despite their ectopic location during ascent, the natural avascular space between the mesosigmoid and the prehypogastric nerve fascia still exists, which lays the anatomical foundation for surgical dissection in this special scenario. The only crossing point of the mesosigmoid and retroperitoneum is the root of the IMA. In the present patient, the procedure of the IMA exposure and resection was relatively easy, as the renal arteries, which originated from the right common iliac artery, were away from the IMA. In one Japanese female diagnosed with rectal cancer coupled with solitary pelvic kidney, the right renal artery branched off from the aortic bifurcation and thought as the IMA. The renal artery was ligated by mistake during laparoscopic surgery, leading to open surgery conversion and vascular anastomosis (6). Therefore, a detailed and precise preoperative CT evaluation of aberrant vessels is of great importance.

The pelvic kidney is sometimes complicated with congenital abnormalities of the genitourinary system. In this patient, the seminal vesicle was replaced by some fiber and fat tissues (Figure 3C). Because of this variation, pelvic anterior dissection was difficult. The seminal vesicle is an important landmark for pelvic anterior dissection. Normally, in males, after cutting the pelvic peritoneum 1 cm above the peritoneal reflection, the seminal vesicle is visible, and dissection should be performed between the seminal vesicle and the posterior layer of the Denonvilliers'fascia until the lower edge of the seminal vesicle is reached, where the posterior layer of the Denonvilliers'fascia should be transected and a deeper surgical plane between the posterior layer of the Denonvilliers'fascia and mesorectum should be entered for subsequent caudal dissection. In the present case, the appearance of tissues in the location where the seminal vesicle should be was similar to that of the rectal wall therefore, a wrong surgical plane was entered. Fortunately, when the ureter and bladder were exposed and identified, we made timely corrections without severe intraoperative complications.

The low rectal tumor in this patient was relatively large and squeezed the mesorectum, leading to suspicion of positive marginal resection fascia (MRF). Additionally, a couple of lymph nodes within the mesorectum were also enlarged and visible. According to National Comprehensive Cancer Network Surveillance Guideline, positive MRF and lymph nodes in low rectal cancer require neoadjuvant radiotherapy. However, for this patient, pelvic irradiation would have an inevitable side effect on the solitary pelvic kidney despite mechanical shielding, resulting in slowly progressive radiation nephropathy or even uremia. Thanks to the radiologists in our multidisciplinary team (MDT), the preoperative tumor staging was precisely decided, and the patient underwent surgery without neoadjuvant therapy. Therefore, the significance of MDT discussion should be valued, particularly for complicated cases.

The present case suggests the feasibility of laparoscopic TME dissection of pelvic solitary kidney in male patients. Comprehensive radiological evaluation, MDT discussion, and careful intraoperative dissection are of great importance.

## Data Availability Statement

The raw data supporting the conclusions of this article will be made available by the authors, without undue reservation.

## Ethics Statement

Ethical review and approval was not required for the study on human participants in accordance with the local legislation and institutional requirements. The patients/participants provided their written informed consent to participate in this study.

## Author Contributions

The authors that contributed to the conception and design of the study were XZ, YD, and YW. Data acquisition and interpretation were performed by XZ, CC, and KW. The first draft of the manuscript was written by XZ. All the authors commented on previous versions of the manuscript and revised it critically for important intellectual content and read and approved the final version of the manuscript to be submitted.

## Funding

This study was supported by the Clinical and Practical New Technology Development Fund of Qilu Hospital of Shandong University (2019-4).

## Author Disclaimer

Informed consent was obtained from the participant for the publication of this case report (including all data and images).

## Conflict of Interest

The authors declare that the research was conducted in the absence of any commercial or financial relationships that could be construed as a potential conflict of interest.

## Publisher's Note

All claims expressed in this article are solely those of the authors and do not necessarily represent those of their affiliated organizations, or those of the publisher, the editors and the reviewers. Any product that may be evaluated in this article, or claim that may be made by its manufacturer, is not guaranteed or endorsed by the publisher.
